# History and complexity in tick-host dynamics: discrepancies between ‘real’ and ‘visible’ tick populations

**DOI:** 10.1186/1756-3305-7-231

**Published:** 2014-05-19

**Authors:** Andrew D M Dobson

**Affiliations:** 1 School of Biological and Environmental Sciences, University of Stirling, Cottrell Building, Stirling FK9 4LA, UK

**Keywords:** Ticks, Tick-host dynamics, Population modelling, Demography

## Abstract

**Background:**

Numerical responses of ticks to changes in densities of their hosts can be complex and apparently unpredictable. Manipulations even of deterministic models can produce counter-intuitive results, including tick populations that either rise or fall under increasing host densities, depending on initial conditions.

**Methods:**

In this paper I use an established simulation model to demonstrate a wide range of numerical responses to different scenarios of host changes, and to examine the basic mechanisms that drive them.

**Results:**

The rate and direction of change of host densities affects the extent to which questing tick numbers reflect those of their hosts. Numerical responses differ profoundly between dynamic tick-host systems and those allowed to reach equilibrium.

**Conclusions:**

The key to understanding tick-host dynamics is to understand the difference between ‘real’ and ‘visible’ tick populations. An appreciation of the implications of this difference – and of the conditions that influence it - will benefit the effective interpretation of field data.

## Background

Among the global suite of arthropod disease vectors that includes mosquitoes, tsetse flies and triatomine bugs, Ixodid ticks present a distinct challenge to epidemiologists. The substantial alterations necessary to adapt the *R*_0_ (basic reproductive number) equation for tick-borne pathogens from that for pathogens vectored by insects [1,2] reflects the many ways in which ticks differ from most insects, including a relatively insignificant degree of mobility, a characteristic of parasitising only one (hard ticks) or very few (soft ticks) hosts per instar, and a long interval between the acquisition and transmission of infective agents.

**Figure 1:**
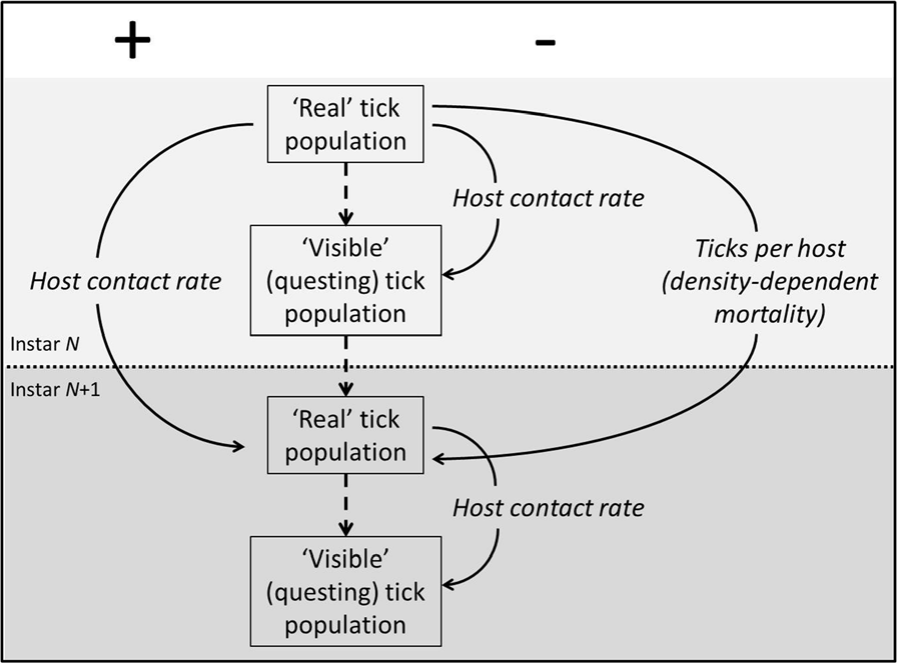
**Host-related influences on ‘real’ and ‘visible’ tick populations (see text for definitions).** Legend: Factors beneath the plus (+) sign act to increase populations, whilst those under the minus (-) sign act to decrease populations. Pale shading denotes instar *N*, darker shading instar *N* + 1.

Ticks transmit a wide array of human pathogens [3,4], and since the discovery and description of Lyme borreliosis in the 1970s – now regarded as the most common vector-borne disease of the northern hemisphere – a huge literature has emerged to describe and catalogue the effects of environmental factors on the abundance of *Ixodes* spp. vectors *inter alia*[5-8]. The potential for error when measuring tick abundance in the field is covered elsewhere [9]; this paper is concerned with the subsequent interpretation of such data. Unfortunately, the abundance of unfed, host-seeking ticks is not a parameter with a straightforward meaning, equivalent to (for example) the abundance of mosquitoes caught in a light-trap. Indeed, the density of questing ticks is merely the visible result of a number of bi-directional and much less visible influences on the ‘real’ density, as outlined in Figure 1, such that the former is only a rough and somewhat variable proxy for the latter. Here, and throughout this paper, I define the ‘real’ population as the underlying baseline of reproductive potential; in this sense, a possible candidate parameter for the ‘real’ density would be ‘the number of egg-laying females per year per unit-area’. Of course, it is extremely unlikely that this would ever be measureable in practice in the field, but insights may be gained from examining the relevant outputs from biological process-based population models, as in this paper. Similarly, I use the terms ‘questing’ and ‘visible’ interchangeably to describe that section of the tick population that may be measured in the field (allowing context to dictate which term is most appropriate).

In short (and leaving aside all considerations of temperature and humidity), where hosts are abundant, ticks are more likely to complete their three blood meals and breed successfully - thereby augmenting the tick population - yet host abundance also determines the rate at which ticks are removed from the vegetation (and hence become unavailable for counting by biologists) [10-12]. The fact that host abundance drives both factors explains the otherwise potentially counter-intuitive observation that the wholesale removal of major host populations initially results in an increase in the questing tick population [13]. The ‘momentum’ of the population, derived from egg-laying females that fed successfully before the hosts were removed, causes the usual emergence of questing ticks, which are then removed from the questing population at a much lower rate than before [10,14]. Regardless of the presence and/or prevalence of alternative hosts, the questing population of larvae in the proceeding generation will therefore be even higher than normal. If one or more of the principal hosts for larvae remain (for example, if deer were the only hosts removed), then some of this population momentum is likely to be maintained through to the nymph stage. The life-history of *Ixodes* ticks, protracted as it is by diapause, means that significant nymphal activity could occur three or even four years after the removal of a major host population. Elsewhere in this paper I shall refer to this surge of questing ticks following host removal as the ‘latent questing population’, and to the extent to which the questing population exceeds levels expected, given current host density, as the ‘degree of latency’.

The contradictory forces of population augmentation (via the provision of meals to reproductive females) and questing tick removal – both imposed by hosts – will result in a variety of outcomes, in terms of questing ticks, under natural conditions. The relative influence of each pressure is likely to be determined by the density-dependent feeding success (perhaps more conveniently thought of as density-dependent mortality) of ticks. Here, density refers to the burden of ticks per host, rather than absolute density of ticks (or hosts) per se, since this mortality is mediated by the individual host’s acquired immune response [15,16] and/or grooming/avoidance behaviour [17].

The fact that density dependence operates at the level of ticks per host means that if a host population steadily increases, the tick population may increase in a similar manner without any great modulating effect of density dependence. Indeed, density-dependent mortality will not rise unless the ratio of ticks to hosts increases. Superficially, as long as the tick population increase is directly caused by a host population increase, the ratio should not be altered. However, in practice, the ratio *does* increase, because the proportion of ticks that successfully feed before dying from moisture stress, predation or disease also goes up, since opportunities to feed become more abundant. Each egg-laying female therefore gives rise to more successfully feeding immature ticks. Whether or not this per-capita increase in survival – and thus greater ratio of ticks to hosts – leads to increased density-dependent mortality will depend upon individual host responses.

Readers should also note the apparent existence of the opposite effect under certain circumstances – density-dependent facilitation, as reported for domestic sheep [18] – but I do not consider this further here.

Density-dependence causes there to exist – theoretically at least - a host-species-specific threshold of host density, above which the real tick population ceases to grow (and may thereafter even decrease, if density-dependence is more than compensatory). Meanwhile, however, the per-tick host-contact rate will continue to increase with increasing host density (unless hosts adopt avoidance behaviour of tick-infested areas, as observed in domestic cattle [19]). At this threshold, recruitment to the questing population is overtaken by removal from it, and the questing population – though not necessarily the real population - will decline [14,20]. Ultimately, density dependence ensures that, for many host species, the relationship between host density and tick density will not be linear across all values of the former [14,20,21].

The processes described above may be summarised as three general phenomena of tick-host population dynamics:

1. There is a difference between ‘real’ and ‘visible’ tick populations, wherein the former is a measure of the reproductive potential (e.g. the number of egg-laying females), and the latter is the number of ticks that are typically available for counting – i.e. the unfed, host-seeking individuals. Both are relevant to considerations of tick-borne disease, but only ‘visible’ populations are measured in the field.

2. Density-dependent host responses to tick infestations may occur.

3. The often-protracted life cycle of Ixodid ticks causes the existence of a temporal lag between changes in the biotic environment and the numerical response of tick populations. This lag ensures that the relationship between host density and tick density is very strongly dependent on the state of the system – i.e. whether the host density is static or changing.

The aim of this paper is to demonstrate the behaviour of tick populations under different scenarios of host density change, and to explain and discuss them with reference to the above three phenomena (which are interconnected to a large extent). The material herein pertains mainly to *Ixodes ricinus*, but many of the principles outlined will be more widely applicable, particularly to the free-living (as opposed to nidiculous) *Ixodes* species, especially *I. persulcatus* in Eurasia and *I. scapularis* and *I. pacificus* in North America.

## Methods

In order to demonstrate a range of tick responses to host population change, outputs were produced from the model of Dobson et al. [22]. This deterministic population model is based around a modified stage-classified Leslie matrix [23], and simulates relative numbers of each tick instar (larva, nymph and adult) in each physiological state according to inputs of biotic and abiotic variables. Simple scenarios of host population change were applied, each of which comprised increasing, constant, then decreasing host densities. Initial host contact rates were taken from the model’s validation site that had the lowest natural values (see [22]) in order to demonstrate the behaviour of the system across the widest possible range of this parameter, and to ensure that the maximum rates reached were still safely within biologically realistic limits. Rates were scaled for graphical purposes such that the initial rate was 1. Climate data from the same site were used.

Changes in large host density were simulated under the following conditions:

*Scenario 1*. Large host contact rate increasing daily over 20 years, reaching a maximum of twice the starting contact rate; holding constant for 20 years; decreasing daily back to the starting contact rate over 20 years; holding constant for 20 years.

*Scenario 2*. Large host contact rate increasing daily over 20 years, reaching a maximum of five times the starting contact rate; holding constant for 20 years; decreasing daily back to the starting contact rate over 20 years; holding constant for 20 years.

*Scenario 3*. Large host contact rate increasing daily over 5 years, reaching a maximum of five times the starting contact rate; holding constant for 20 years; decreasing daily back to the starting contact rate over five years; holding constant for 20 years.

*Scenario 4*. Large host contact rate increasing daily over 5 years, reaching a maximum of twice the starting contact rate; holding constant for 20 years; decreasing daily back to the starting contact rate over five years; holding constant for 20 years.

**Figure 2:**
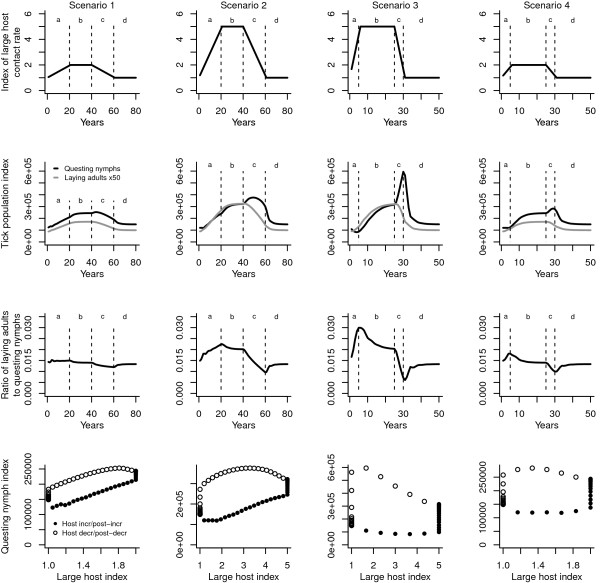
**Outputs from simulations of the tick population model under four scenarios of large host contact rate change (see text for details).** Legend: Simulations were run for either 80 (Scenarios 1 and 2) or 50 (Scenarios 3 and 4) years. The top row shows the rate and extent of change of host contact rate in each scenario. The second row shows annual AUTG values (see text) for questing nymphs (black lines) and egg-laying adult females (multiplied by 50 for aid of visual interpretation; grey lines). The third row shows the ratio of laying adult to questing nymph AUTG. In the first three rows, the periods of host increase, stability, decrease and stability are delineated with vertical dashed lines, and marked a, b, c and d, respectively, for clarity. The fourth row shows scatterplots of questing nymph AUTG against the large host contact rate index (points from phases of the simulation when the host index was increasing, or stable following an increase, are shown as closed circles; points from phases of decrease, or post-decrease stability, are shown as open circles).

All scenarios were preceded by a ten-year run of initial conditions to ‘bed-in’ the tick population [14]. Results, including schematics of host change scenarios, are shown in Figure 2.

In these plots, the questing nymph index is calculated by summing the daily questing abundance across the year (described elsewhere as the area under the graph of questing ticks, or AUTG [9]). (Though I do not directly discuss the implications, the reader should note that individual ticks will be counted more than once in this metric – which is equivalent to the seasonal questing profile derived from field studies - and that the lower the host contact rate, the more times an individual tick will be counted, since there is a lower likelihood that it will have been removed from the questing population since the previous day).

**Figure 3:**
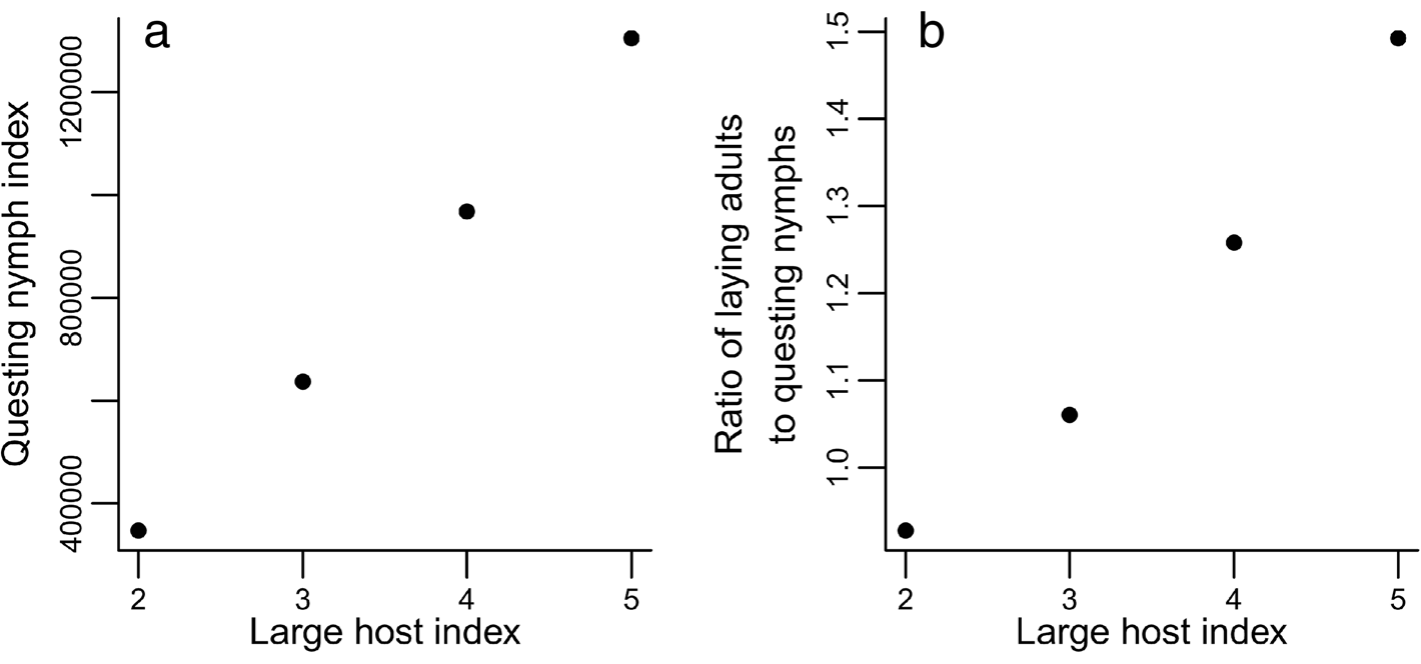
**Equilibrium tick population abundances from four simulations of the tick population model where populations experienced constant large host contact rates of different magnitudes.** Legend: **(a)** Questing nymph index. **(b)** Ratio of laying adults to questing nymphs.

One of the aims of this paper was to identify the difference in the tick-host relationship between dynamic (i.e. with changing host contact rates) and static (with constant host contact rates) systems. For this reason, simulations were also performed where the large host contact rate was held constant at levels of 2, 3, 4 and 5 times the starting rate, and the models were run until tick populations reached equilibrium. Selected results are shown in Figure 3.

## Results and discussion

The plots in Figure 2 illustrate various points of commonality and difference between the effects of the host-change scenarios, which are discussed in turn.

### Variable relationships between questing nymphs and host contact rate

The undoubted truisms that (i) lots of hosts mean lots of ticks, and (ii) a lack of hosts means no ticks, nonetheless lie at very simple extremes that are linked by anything but a simple continuum of intermediates. A cursory glance at all plots in the fourth row of Figure 2 would suggest that there is no clear relationship between host density and tick density. However, Figure 3a demonstrates very clearly that the relationship is simple and linear over the same range of host contact rates if one looks at final densities of tick populations that have been allowed to reach equilibrium. (Here, densities are not yet high enough to trigger a density-dependent response).

This difference is critical – populations at equilibrium behave very differently from those that have not yet reached it, and the rate and direction of change in host density are both influential. The scatterplot points of the fourth row of plots in Figure 2 have been separated into those that derive from years in the simulations when host contact rates are increasing or immediately post-increase (closed circles), and those where the rates are decreasing or post-decrease (open circles). Identifying the derivation of these points – the ‘population history’, if such a term is helpful - is the first step in understanding the underlying mechanisms. The patterns and processes described below should provide a context in which these plots become less surprising than they might otherwise be.

### A rising nymph population with a declining host contact rate

The sudden loss of a host of adult ticks is often followed by a surge in questing in the next few years as the momentum of the tick population carries through before fading (e.g. [13]). Results from Scenario 2 show that if the host population is steadily reduced, rather than suddenly removed, this surge in questing ticks may be relatively long-lasting (approximately ten years, in this case). Indeed, the rate of host removal has a direct impact on the magnitude of the surge in questing ticks, as can be seen by comparing the second row of plots in Figure 2 between Scenario pairs 2 & 3, and 1 & 4. In both cases, a reduction in host contact rate of the same extent, but different speed, results in questing nymph peaks of differing magnitudes. The explanation is largely quite simple and mechanical; the faster the reduction in host contact rate, the smaller the proportion of the latent questing population gets removed by hosts from the vegetation, leaving more to be counted. The population momentum is thus more apparent when the host decline is steeper.

Special variants of this general phenomenon appear to operate when major hosts are only removed/excluded from part of a larger area infested with ticks. As one should predict, experimental exclosures that prevent entry of deer to field sites are subject to ‘edge effects’ that mask, to a greater or lesser extent, the effect of having removed a major host of the reproductive stage of ticks [24,25]. Some studies report increases in tick activity in exclosures relative to control plots outside, even after sufficient time has elapsed to nullify the effects of the population momentum described above. This putative phenomenon has significant implications for epidemiology, and merits further investigation. In the only meta-analysis to address questing tick amplification in exclosures, Perkins et al. [26] argued that there was a threshold area for deer exclosures (about 2.5 ha), below which amplification occurred. However, there are at least two good reasons to be wary of the empirical data. First, the four data points underlying the above estimation of the threshold area represent varying combinations of three instars of two species from different genera (*Amblyomma americanum* and *I. scapularis*), when it might be expected that different instars and different species would display distinct responses to deer removal. Secondly, three of the four data points are derived from studies that are either partly (*N* = 2) or entirely (*N* = 1) based on exclosures established within 1 or 2 years of tick data collection. This leaves the data extremely vulnerable to the effects of population momentum, and hence not indicative of the long- or even medium-term effect of exclosure. Nonetheless, the literature does offer more than one example of such apparent amplification (e.g. [24]), so its occurrence needs to be explained.

An amplification in exclosures should be counterintuitive if one starts with the basic assumption that tick populations require a host to feed the reproductive stage (i.e. adult females), and that deer and other large herbivores typically fulfil this role; relatively few adult ticks feed successfully on rodents under natural conditions [27,28]. Of course, it should be quite quickly apparent that the movement of rodents across exclosure fences will allow for the ‘importation’ of engorged larvae that will later quest as nymphs within the exclosure (even if the vast majority that end up as questing adults in the exclosure will ultimately fail to reproduce); however, a further explanation is required to account for actual increases in – not merely persistence of - questing nymphs relative to the outside in small exclosures.

The most parsimonious mechanism for the amplification of ticks in exclosures relative to the unfenced outside is a form of constantly maintained population momentum (which is separate from the initial surge of questing ticks that will have followed immediately upon the erection of exclosure fencing). Pugliese & Rosà [21] provided a mathematical demonstration of the phenomenon (though they treated ticks per host rather than questing ticks as the variable of interest), but a brief – and non-mathematical - explanation here is appropriate.

There are fewer hosts inside an exclosure than outside, so there are more engorged ticks deposited in a given unit area outside exclosures than inside. Hosts that are able to cross the fence – e.g. rodents and small birds - import and export engorged ticks. After development to the next stage, ticks dropped in exclosures are less likely to be picked up by hosts than those that were dropped outside, meaning that they are more likely to be available for counting at any given time. There are therefore two pressures acting in opposite directions – low host numbers inside exclosures mean fewer opportunities to feed, but also a greater likelihood of being counted – a higher degree of ‘latency’. With no movement of hosts across exclosure fences, the tick population either side would be broadly proportional to host density. However, this situation begins to change if hosts move across fences, since the import of ticks should exceed export (given that hosts moving out of an exclosure will likely have picked up fewer ticks than those moving in, assuming initially higher densities of ticks outside). Exported ticks are more likely to find hosts than imported ticks, because of higher host densities outside, so the number of questing ticks available to be counted in exclosures will increase relative to that outside. Tick densities in exclosures can continue to rise relative to the outside even if import/export becomes balanced, as long as overall host densities remain lower in exclosures, since this will result in more unfed ticks in exclosures.

Amplification in exclosures is therefore theoretically feasible, but Perkins et al.’s [26] suggestion of a general threshold area below which it occurs is unrealistic, on the same, purely theoretical grounds. Indeed, the extent of amplification of questing tick numbers in exclosures ought to be dependent upon four factors, the first two of which – degree of fence-crossing by small hosts and the exclosure size – are inversely related to one another and can be considered as alternative, reciprocal measures of the same fundamental process [21]. The smaller the exclosure and the greater the degree of fence-crossing, the greater the potential for import of engorged ticks which will then produce questing ticks of the next stage that are less likely to be removed by hosts (higher latency) than those that were dropped outside.

The third factor is density dependence. As mentioned in the Introduction, the strength of this effect determines the point of balance between the two host-mediated forces - population augmentation (via the provision of meals to reproductive females) and questing tick reduction (via successful feeding and therefore removal from the questing population). To re-cap: Inside the exclosure, removal by hosts is lower (because there are fewer hosts), but augmentation via successfully fed adults is also lower (because adults have fewer feeding opportunities). When density-dependent mortality is operating (i.e. when lower host densities mean higher per-tick feeding success inside than outside), the population augmentation force becomes relatively less dominant than the questing tick removal force. The discrepancy in host density between inside and outside therefore has less influence on tick reproduction, resulting in a further relative increase in questing numbers within the exclosure.

In their two-host system, Pugliese & Rosà [21] also demonstrated an effect of the fourth factor, the contact rate between ticks and the larger (i.e. non-fence-crossing) hosts; the higher the large host contact rate, the higher the likelihood of amplification inside exclosures. This parameter is better thought of as the ratio of tick-host contact rate inside and outside exclosures; since large hosts do not enter exclosures, raising the large host contact rate means that the host contact rate overall becomes relatively higher outside, but is unchanged inside. This causes greater tick population augmentation outside, and hence a greater degree of engorged tick import into exclosures, whilst simultaneously increasing unfed tick removal outside (masking the population augmentation) without altering it inside (thereby allowing imported ticks to result in greater questing numbers).

It should be obvious, therefore, that whilst there ought to be a threshold point for any scenario, above which exclosures show amplified tick densities, this threshold will vary according to the exclosure area, degree of fence-crossing, nature of density dependence and ratio of host contact rates inside and outside. The shape of the exclosure (and more specifically, the edge-to-area ratio) should also be influential, since it will affect patterns of fence-crossing and the spatial distribution of ticks across the exclosure.

### ‘Real’ versus ‘visible’ populations

Comparison of the laying adult and questing nymph plots in the second row of Figure 2 indicates that the number of laying adults follows the host contact rate (top row of plots) in a far more consistent manner than does the number of questing nymphs, particularly in the time period marked ‘c’. All other things being equal (as they are in these simulations), host contact rate should drive the tick population in a dominant manner – with some discrepancy due to the effects of density dependence – so the broad agreement between laying adults and host contact rate confirms that the former represents a good metric of the ‘real’ population (as opposed to the ‘visible’, questing population). This also means that the ratio of laying adults to questing nymphs (third row of plots) is a function of the discrepancy between real and questing populations. The ratio provides another way of interpreting and rationalising the peak in questing nymphs that continues as host contact rates decline (sections ‘c’, second row of plots). Across all scenarios, the size of this nymphal peak is proportional to the magnitude of the change in the ratio of laying adults to questing nymphs during the earlier phase of host density increase (sections ‘a’, third row of plots). In other words, the disparity between real and questing populations gives a measure of the population’s ‘capital’, which is released as population momentum when host densities decrease. Thinking in these terms helps to explain why the questing nymph peak is higher when the host contact rate decline is faster; the capital is released over a much shorter time period in Scenarios 3 & 4 than in 1 & 2, hence the consequent surge of questing ticks is more acute.

**Figure 4:**
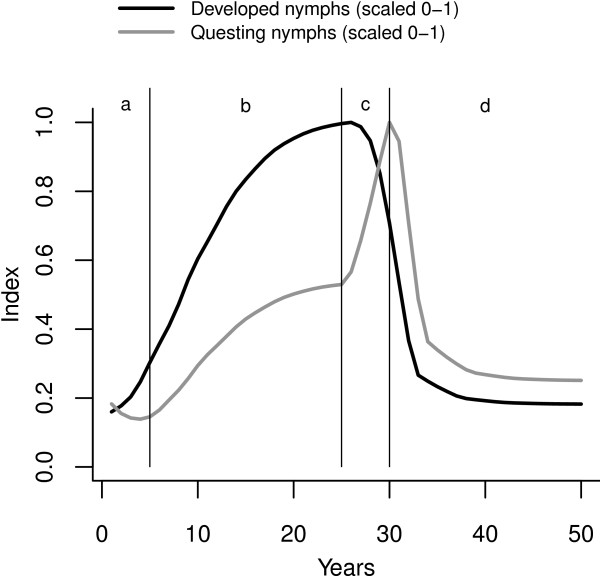
**‘Real’ and ‘visible’ populations from Scenario 3 model simulations.** Legend: Real: Developed nymphs prior to questing (black line); Visible: Questing nymphs (grey line).

The broad similarity between plots in the first and third rows of Figure 2 shows that the higher the host contact rate, the greater the proportion of the real population that is hidden from the view of blanket-dragging biologists. This relationship holds true when populations are allowed to reach equilibrium, as in Figure 3b, where the ratio between laying adults and questing nymphs increases (slightly more than) linearly with host density. In Figure 4 the distinction between real and visible populations is made more explicit; the questing (visible) nymph population is plotted next to the nymphs that have completed development and are ready to quest. The latter is a good representation of the real population in this context, but is unavailable for measurement by the field biologist.

### Synthesis and applications

“All models are wrong, but some are useful” [29]; here, population models are useful because they give us the opportunity to investigate demographic processes in a way that is effectively impossible in an ordinary empirical study. The model of Dobson et al. [22] is among a small number of models that attempt to capture the convoluted life-history of *Ixodes* spp. and formally characterise population processes in simple mathematical terms. Providing that they are structured in a manner that sufficiently reflects the realities of the system they aim to simulate, these models may be thought of as strictly controlled experimental environments in which the effects of individual factors may be assessed in isolation [14]. Here, this rarefied experimental environment has illuminated conditions that we must take into account when interpreting tick field data - namely the influence of ‘population history’, whereby the direction of response to host density change may be influenced by previous, unmeasured states.

The dominant issue outlined in this paper – the fact that reliable measurement of questing ticks will not necessarily lead to a reliable understanding of the population under study – is not one that may be practically resolved in the field. Questing ticks can be accurately surveyed by blanket dragging, assuming that an appropriate sampling regime is employed [9], and there are various techniques for measuring the abundance of different host species, but there is no realistic way of recording the density of laying adult female ticks without going through the extremely laborious and inaccurate process of wholesale extraction of large samples of vegetation and topsoil and picking through it to look for them.

It is possible that some insight into the problems caused by population history may be gained from looking at precise patterns of questing ticks and those on hosts through the year, without reverting to direct examination of ‘real’ population size. Any mechanistically realistic population model would allow detailed examination of these variables and thereby potentially aid the correct interpretation of confusing field data; whether or not enough empirical data yet exist for model parameterisation remains to be seen. Models that explicitly incorporate host individuals (as opposed to host contact rates for tick individuals) would be capable of revealing the epidemiological implications of different values of the ratio of laying adults to questing nymphs (i.e. the ratio of real to visible population size), and should be a focus for future study.

One facet of this particular population model may not be universally applicable, and would benefit from further empirical research: Density dependence is here depicted as a linear response, after a certain threshold has been reached. This is not necessarily realistic. If tick feeding were highly clustered on a few hosts, a threshold model of host response could result in greatly over-compensating density dependence, and possibly fluctuating tick population dynamics, at almost any value of tick-host ratio. It is hoped that future incarnations of the population model will incorporate such mechanisms, following additional empirical input.

In many cases, of course, a given tick population under natural conditions will not experience great fluctuations in numbers of its hosts (the obvious exception being the cyclic dynamics of microtine rodents), meaning that most differences in population size over time will be determined more by a subtle shifting of the host community structure - or some abiotic factor – than by the sorts of processes outlined here. However, the majority of tick-borne disease research interest is currently focussed upon areas where ecological change is, or is predicted to be, extensive – especially regarding the question of whether biodiversity loss will amplify the density of infected ticks [30-32]. Data from field sites where host densities have not been stable for at least ten years may lead researchers to erroneous conclusions if they do not have an appreciation of the considerable complexity of tick population biology. Specifically, tick biologists must acknowledge that numerical responses of ticks to changes in host density are not necessarily consistent between populations. As a consequence, it will be almost impossible to infer much information from ‘snapshot’ sampling of a tick population and those of its hosts, especially if any recent change is suspected in the latter. It is also fundamentally important to recognise that data points drawn from the same population in different years are unlikely to be statistically independent unless the temporal separation is large. Likewise, a set of tick abundance samples taken simultaneously from several separate sites may not be meaningfully related to the corresponding set of host density (or meteorological) data.

## Conclusions

Tick population biology is complex. Changes in host density may have counterintuitive effects on the abundance of questing ticks; the key to understanding these numerical responses is to appreciate the difference between real and visible tick populations. An understanding of the suite of mechanisms that drive tick population dynamics – and the range of outcomes they may produce – will aid interpretation of data from field studies, and should warn against over-extrapolation from any particular set of results. Further research on epidemiological implications is required.

## Competing interest

The author declares that he has no competing interests.

## Authors’ contributions

ADMD conceived and designed the study, analysed the data and wrote the manuscript.
